# From explainability to clinical actionability: translating artificial intelligence models into decision support for endocrine disease management

**DOI:** 10.3389/fendo.2026.1887643

**Published:** 2026-08-21

**Authors:** Tianqiang Wu, Wenpin Cai, Zhixiang Li

**Affiliations:** 1 Wenzhou TCM Hospital Affiliated to Zhejiang Chinese Medical University, Wenzhou, China; 2 Department of Traditional Chinese Medicine, Xuzhou Central Hospital, Southeast University, Xuzhou, China

**Keywords:** artificial intelligence, clinical decision support, diabetic kidney disease, explainable AI, GLP-1 receptor agonists, obesity, polycystic ovary syndrome, thyroid disease

## Abstract

Artificial intelligence (AI) models are increasingly reported in endocrine disease management, but clinical value cannot be inferred from discrimination, model complexity, or explainability alone. This narrative review synthesizes evidence from diabetes and diabetic kidney disease (DKD), thyroid disease, polycystic ovary syndrome (PCOS; with attention to the proposed polyendocrine metabolic ovarian syndrome, PMOS, terminology transition), and obesity/GLP-1-based metabolic management through an actionability framework. We define clinical actionability as the link between a meaningful endocrine prediction and transparent reporting, bias and applicability appraisal, external or temporal validation, calibration, threshold-based utility, interpretable output, workflow integration, fairness assessment, and post-deployment monitoring. Diabetes/DKD provides a comparatively broad evidence base, including decision-support examples, non-invasive triage, calibration, decision-curve analysis, and early deployment-oriented work. Thyroid AI increasingly reports validation and clinical-utility metrics for nodule triage, biopsy decisions, and risk prediction, but prospective workflow evidence remains limited. PCOS/PMOS AI addresses diagnostic and reproductive-endocrine gaps, yet requires broader phenotype alignment, fairness analysis, and multi-setting validation during the PMOS terminology transition. Obesity/GLP-1 evidence now includes response-prediction, benefit-stratification, treatment-intensification, persistence, and remote-care examples, but it does not establish AI-guided treatment selection, adherence/tolerability prediction, or monitoring. Across modules, differences lie less in headline discrimination metrics, including the area under the receiver operating characteristic curve (AUC), than in validation, utility, workflow, fairness, and monitoring evidence. Endocrine AI should move from explainable prediction toward decision support that can be interpreted, thresholded, acted on, monitored, and revised in clinical workflows.

## 1 Introduction: why endocrine AI needs an actionability lens

**Figure 1:**
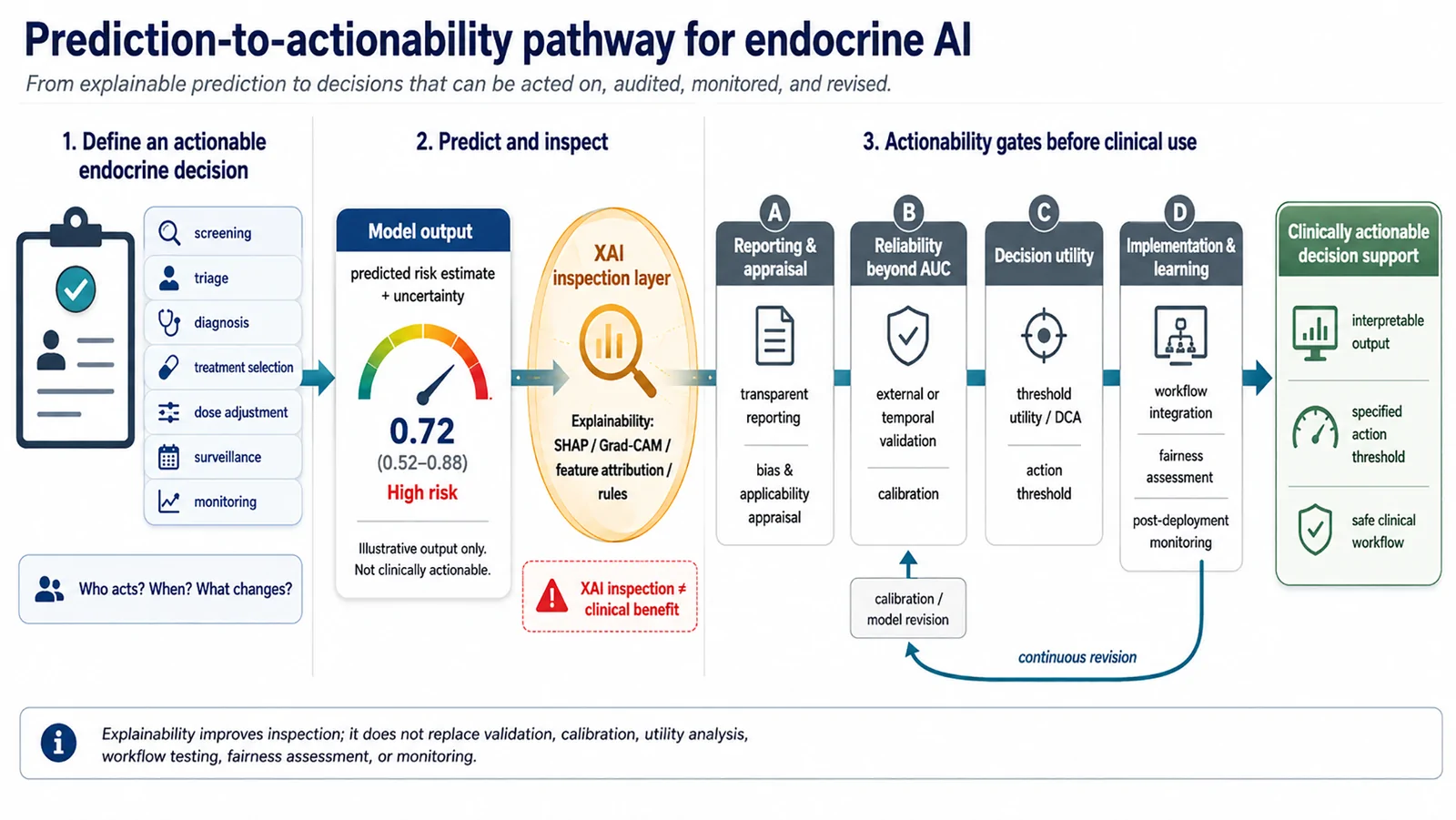
Prediction-to-actionability pathway for endocrine AI. The image presents the proposed pathway from an actionable endocrine decision to model-assisted decision support. A clinically meaningful problem first has to be linked to a specific action, such as screening, triage, diagnosis, treatment selection, dose adjustment, surveillance, or monitoring. The model output is shown as a predicted risk estimate with uncertainty, followed by an XAI inspection layer using methods such as SHAP, Grad-CAM, feature attribution, or rules. Before clinical use, the output should pass actionability gates covering reporting and appraisal, reliability beyond AUC, decision utility, and implementation and learning, including transparent reporting, bias and applicability appraisal, external or temporal validation, calibration, threshold utility or DCA, workflow integration, fairness assessment, post-deployment monitoring, and revision. The image emphasizes that XAI inspection is not equivalent to clinical benefit and does not replace validation, utility analysis, workflow testing, fairness assessment, or monitoring.

Artificial intelligence is increasingly being developed for endocrine care, including prediction, screening, treatment support, and longitudinal monitoring in diabetes, diabetic kidney disease, thyroid disease, PCOS/PMOS, and obesity-related metabolic management. Recent diabetes-focused reviews cover predictive analytics, clinical decision support, and generative AI (1–3), while endocrine-wide mapping and obesity/GLP-1-focused work point to individualized risk and treatment-response frameworks (4, 5). In practice, the actionability question may be whether a patient with type 2 diabetes should enter intensified kidney surveillance, whether a thyroid nodule should proceed to fine-needle aspiration biopsy, or whether an early GLP-1 response pattern should support closer follow-up rather than a premature claim of AI-guided treatment selection. **Figure 1** summarizes this prediction-to-actionability pathway. Across these examples, the central translational question is no longer whether an algorithm can produce a risk score or an explanation. It is whether the output can safely change a clinical decision.

Existing reviews and model-development papers document expansion in endocrine and metabolic AI, but they usually organize the literature by disease area, algorithm family, or discrimination metric rather than by translational readiness. Methodological guidance for AI decision support, clinical prediction reporting, and model utility emphasizes that performance claims should be judged together with reporting quality, applicability, calibration, threshold utility, and clinical evaluation (6–8). This gap, and the recurring methodological limitations summarized in Section 2.6, motivate the actionability-based synthesis developed here.

This is a focused narrative, framework-oriented review rather than a systematic review or meta-analysis. The evidence set was assembled from PubMed and OpenAlex searches run on May 18, 2026, supplemented by targeted citation checking, DOI/PMID/Crossref verification, and full-text or abstract-level checks through May 20, 2026. Search themes combined endocrine AI, explainable AI, clinical decision support, actionability, validation, calibration, decision-curve analysis, workflow, fairness, monitoring, and the four disease modules. Study selection was purposive and iterative: priority was given to recent reviews for module framing and to original studies that illustrated at least one actionability domain. Because the review was purposive and framework-oriented, no PRISMA flow diagram was generated, and no formal study-level risk-of-bias synthesis or quantitative meta-analysis was undertaken. The synthesis is qualitative and comparative rather than exhaustive.

Endocrine diseases usually require repeated decisions over time rather than a single diagnostic act. Glycemic titration, kidney-risk surveillance, thyroid nodule triage, reproductive-endocrine diagnosis, and weight-management personalization all depend on thresholds, trade-offs, patient context, and follow-up workflows. A DKD model, for example, is actionable only if the predicted risk can be calibrated to a surveillance or referral threshold that clinicians can use; similarly, a thyroid nodule classifier becomes clinically relevant only when its risk estimate can be mapped to biopsy, observation, or surgical pathways. A model that performs well in a development cohort may still be unusable if its risk estimates are poorly calibrated, if the action threshold is unclear, if the explanation cannot be integrated into clinician reasoning, or if performance drifts after deployment (6–8).

Explainability is necessary but insufficient. Shapley additive explanations (SHAP) values, feature-importance rankings, Grad-CAM heatmaps, or other explanation layers can help inspect model behavior, communicate influential predictors, and identify potential failure modes. However, these outputs do not by themselves establish clinical utility, transportability, fairness, or patient benefit. For this review, clinical actionability is defined as the degree to which an AI model links a clinically meaningful prediction to reliable evaluation, interpretable and usable outputs, a specified decision threshold or action, workflow integration, fairness assessment, and post-deployment monitoring.

The aim of this narrative review is to evaluate when endocrine AI moves beyond explainable prediction to clinically actionable decision support. Specifically, we compare diabetes and diabetic kidney disease (DKD), thyroid disease, polycystic ovary syndrome (PCOS), including the proposed polyendocrine metabolic ovarian syndrome (PMOS) terminology transition, and obesity/GLP-1-related metabolic management across six translational domains: clinical task linkage, external or temporal validation, calibration and decision utility, workflow integration, fairness assessment, and post-deployment monitoring. Because most source studies, datasets, and clinical labels still use PCOS, this review uses PCOS as the default term and refers to PMOS when discussing the proposed multisystem terminology shift or papers that use that framing (9). The motivation is that clinicians, reviewers, and health systems need to know whether model outputs can safely change real decisions, not simply whether a model is explainable or reports a high AUC. The scope is therefore framework-oriented and comparative: it uses four endocrine modules to make cross-disease actionability differences visible and to provide practical appraisal questions for authors, clinicians, reviewers, and editors working at the interface of endocrine AI and decision support.

**Figure 2:**
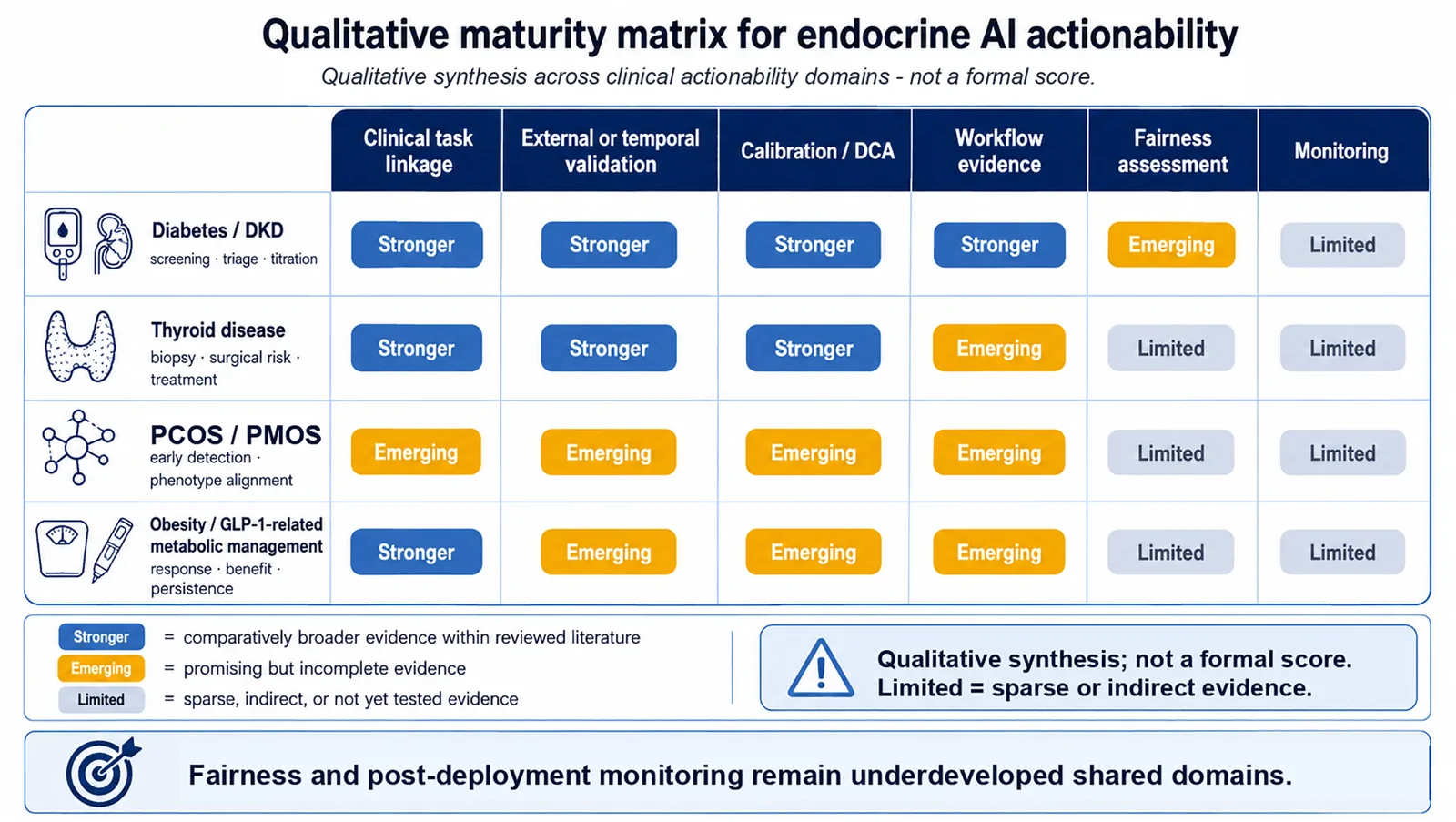
Qualitative maturity matrix for endocrine AI actionability. The image provides a qualitative comparison of diabetes/DKD, thyroid disease, PCOS/PMOS, and obesity/GLP-1-related metabolic management across six actionability domains: clinical task linkage, external or temporal validation, calibration/DCA, workflow evidence, fairness assessment, and monitoring. The row annotations highlight representative clinical tasks, including screening, triage, titration, biopsy, surgical risk, treatment, early detection, phenotype alignment, treatment response, benefit, and persistence. Domain labels indicate where evidence is comparatively broader, emerging, or limited within the reviewed literature. Limited indicates sparse, indirect, or not yet tested evidence, not absence of evidence. The matrix is intended as a qualitative appraisal aid rather than a formal scoring system and highlights fairness and post-deployment monitoring as underdeveloped shared domains.

This Review does not provide another disease-by-disease catalogue of endocrine AI. Its contribution is to apply one gate-based actionability framework across four endocrine modules, use **Figure 2** as a qualitative maturity matrix, and convert the comparison into reusable appraisal questions in **Table 1**. This structure makes it possible to compare why diabetes/DKD currently has a comparatively broad evidence base, why thyroid AI is moving beyond classification toward external validation and utility metrics, why PCOS/PMOS and obesity/GLP-1 remain clinically important but uneven, and why fairness and post-deployment monitoring remain shared underdeveloped domains (10, 11).

**Table 1:** Practical appraisal questions for endocrine AI manuscripts.

Appraisal domain	Evidence to look for	Why it matters for endocrine AI	Common failure mode	Cautious wording
Clinical task and action	Intended use, target user, prediction timing, and possible action	Endocrine care depends on thresholds, follow-up intervals, treatment adjustment, and referral decisions	Reporting a model endpoint without stating what decision should change	“The model may support risk stratification or triage in a defined setting.”
Reporting and bias appraisal	Transparent predictors, outcome definitions, missing-data handling, validation design, and risk-of-bias/applicability assessment	A model cannot be interpreted or transported if the development process is unclear	Treating high discrimination as sufficient despite unclear sampling, leakage, or predictor availability	“The model is reported sufficiently for appraisal, but clinical use requires further validation.”
External or temporal validation	Evaluation in independent sites, time periods, devices, assays, or health systems	Diabetes, thyroid, PCOS/PMOS, and obesity datasets vary by care pathway, phenotype, and measurement context	Internal validation only, or external validation without calibration review	“External or temporal validation supports transportability but does not by itself prove outcome benefit.”
Calibration and threshold utility	Calibration plots or metrics, clinically plausible thresholds, and DCA or net-benefit analysis	Many endocrine decisions depend on absolute risk and action thresholds rather than ranking alone	Reporting AUC without showing whether predicted risks support action	“Calibration and threshold-based utility should be assessed before decision support is claimed.”
Explainability	SHAP, Grad-CAM, feature attribution, nomogram, rules, or clinician-understandable explanation linked to a decision	XAI can help inspect model behavior, reveal implausible predictors, and support communication	Presenting explanation plots as evidence of clinical safety or benefit	“Explainability improves inspection; it is not proof of clinical benefit.”
Workflow and usability	Where the output appears, who acts on it, comparator workflow, user understanding, alert burden, and fail-safe plan	Endocrine AI must fit repeated clinical decisions rather than add unsafe automation burden	Web apps or calculators shown without testing real workflow effects	“Workflow integration remains preliminary unless evaluated with intended users.”
Fairness and monitoring	Subgroup performance, subgroup calibration, drift monitoring, retraining/recalibration triggers, and post-deployment audit	Endocrine phenotypes, treatment access, devices, assays, and follow-up intensity differ across populations	No subgroup analysis, no monitoring plan, or no drift response strategy	“Fairness and monitoring should be planned before deployment and reassessed after implementation.”

This table summarizes the main domains used in this review to judge clinical actionability, including evidence to look for, why each domain matters, common failure modes, and suggested cautious wording for manuscript claims. It is intended as an appraisal aid rather than a formal clinical guideline.

## 2 From prediction to actionability: a cross-disease evaluation framework

### 2.1 Defining actionability

In this review, actionability is not treated as a synonym for prediction accuracy. A clinically actionable endocrine AI system should define the clinical problem, identify the decision that may change, report the model transparently, undergo risk-of-bias and applicability appraisal, demonstrate reliable performance beyond discrimination, provide interpretable outputs, and specify how its recommendation enters a real workflow. For example, whether a thyroid nodule should proceed to fine-needle aspiration depends not on the AUC of a classifier alone, but on whether a calibrated risk crosses a biopsy threshold within the clinician’s workflow. This definition is aligned with reporting and evaluation guidance for prediction models and AI decision-support systems, but it is applied here to the particular decision structure of endocrine care (6, 12).

The framework is intentionally gate-based. The first gate asks whether the clinical task is explicit: screening, diagnosis, prognosis, treatment selection, monitoring, or workflow prioritization. The second asks whether the data, predictors, outcome definition, missing-data handling, and model-development process are sufficiently reported and appraised. The third asks whether performance has been evaluated in a way that matters for action, including external or temporal validation, calibration, and threshold-based utility. In practical terms, the analytic schema is input -> actionability gates -> decision-support output: predictors and model estimates are not considered clinically meaningful until they pass through checks that define who acts, when action is triggered, and what comparator workflow is being improved. Only after these gates does the explanation layer become clinically meaningful.

### 2.2 Reporting, bias, and applicability before clinical claims

Transparent reporting and bias appraisal are early checks because a model cannot be responsibly interpreted if its data source, predictor handling, outcome definition, and validation design are unclear. TRIPOD+AI supports structured reporting for clinical prediction models using regression or machine-learning methods, while PROBAST+AI provides a dedicated framework for risk-of-bias and applicability assessment in prediction models using regression or AI methods (12, 13). These tools do not prove that a model is useful; rather, they help determine whether a model can be judged at all.

This distinction matters in endocrine AI because many studies are retrospective, single-center, or based on datasets collected for purposes other than the proposed clinical action. In such settings, a high AUC may coexist with unclear patient selection, unstable outcome definitions, hidden leakage, limited transportability, or predictor availability problems. Reporting and appraisal tools should therefore be viewed as the entrance to actionability, not as a substitute for clinical utility.

### 2.3 Calibration, thresholds, and decision-curve analysis

Discrimination answers whether higher-risk patients tend to receive higher scores than lower-risk patients; it does not answer whether an individual patient’s estimated risk is accurate enough to guide a threshold-based decision. Calibration is therefore central to endocrine AI, where decisions often depend on absolute risk, treatment thresholds, surveillance intervals, or referral triggers. A poorly calibrated model can look impressive by AUC while still misclassifying the urgency or intensity of clinical action (7).

Decision-curve analysis (DCA) adds a complementary question: whether using a model at plausible threshold probabilities would provide net benefit compared with treating all patients, treating none, or following usual strategies. This is particularly relevant for endocrine decisions such as biopsy triage, kidney-risk screening, hypoglycemia prevention, diabetic-retinopathy referral, or treatment-response prediction. DCA should not be treated as definitive implementation evidence, but it moves evaluation closer to the decision that the model is supposed to support (8).

### 2.4 Explainability as inspection, not proof of benefit

Explainability methods can make endocrine AI more inspectable, but the review treats them as an intermediate layer rather than an endpoint. Feature-attribution methods can identify predictors that drive a score, image explanations can localize regions that influence classification, and rule-based or staged decision-support systems can expose parts of the reasoning process. These outputs may improve clinician trust or reveal implausible behavior, yet they do not establish that a model improves decisions, reduces harm, or generalizes across settings (6, 14).

This distinction should shape how disease papers are written about. A study that reports SHAP values, Grad-CAM heatmaps, or a web calculator may be more transparent than a black-box model, but it remains clinically preliminary unless the output is connected to a decision threshold, evaluated in external or temporal data, tested against relevant comparators, and situated in a workflow. Web tools and calculators should therefore be described as implementation signals or prototypes, not as evidence of outcome benefit unless prospective use has been evaluated.

The choice of model class itself involves an interpretability-performance trade-off that is not unique to medicine: intrinsically transparent models such as single decision trees are easy to inspect but can be outperformed by ensemble methods such as random forests, a pattern documented even in non-clinical machine-learning tasks (15). A recent non-endocrine medical-imaging example makes the same boundary visible: a brain-tumor MRI model reported 99.5% accuracy, Grad-CAM visualization, and ablation analyses, but these remain model-evaluation signals rather than evidence of endocrine workflow benefit (16). In endocrine AI, this reinforces that interpretability and model class should be evaluated against the clinical decision rather than assumed, because neither a more transparent nor a more complex model is automatically more actionable.

### 2.5 Workflow, fairness, and monitoring

The final checks concern what happens when an AI system leaves the development dataset. Workflow fit asks whether the model output reaches the right clinician or patient at the right moment, whether the recommendation is understandable, and whether it changes work without creating unsafe automation bias. Fairness asks whether performance, calibration, or clinical consequences differ across sex, age, ethnicity, socioeconomic status, disease subtype, or care setting. Monitoring asks whether performance is tracked after deployment as populations, devices, assays, workflows, and treatment patterns change (17, 18).

These gates remain less mature in the endocrine literature than discrimination, XAI, or even calibration. Some recent work, such as deployment-oriented DKD prediction, has begun to report subgroup fairness and web-app implementation, but this remains the exception rather than the rule in the papers reviewed here (19). The implication is not that endocrine AI should wait for perfect evidence before all clinical experimentation. Rather, early implementation should be evaluated as implementation, with explicit attention to safety, drift, equity, clinician reliance, and monitoring.

**Figure 1** summarizes this framework as a prediction-to-actionability pathway for endocrine AI. It links an actionable endocrine decision to a model output with uncertainty, an XAI inspection layer, four actionability gate families before clinical use, and clinically actionable decision support, with continuous revision through calibration, monitoring, and model updating. It is intended to make the review’s central distinction explicit: explainable prediction is an intermediate step, whereas clinical actionability requires reporting, reliability, utility, workflow integration, fairness assessment, monitoring, and revision.

### 2.6 Recurring limitations of existing endocrine AI models

Across the reviewed endocrine AI literature, several limitations recur across disease modules. Many studies are retrospective, single-center, or based on opportunistic clinical datasets; external or temporal validation is inconsistent; calibration and DCA are less frequently reported than discrimination; and decision thresholds are often not mapped to a specified clinical action. PCOS/PMOS studies raise additional concerns about phenotype heterogeneity, label transition, dataset size, class imbalance, and possible leakage, while obesity/GLP-1 studies often remain observational and do not yet test AI-guided treatment selection prospectively (10, 11).

**Supplementary Table 1** makes these limitations visible at study level. Rather than functioning as a formal risk-of-bias tool or meta-analysis, it compares representative source studies by clinical task, reported performance, design, validation type, calibration, DCA, XAI method, workflow evidence, fairness or subgroup assessment, monitoring, and key limitation. This structured comparison provides a review-appropriate response to questions about evaluation under different conditions without implying that this article conducted new ablation experiments or *de novo* statistical testing.

## 3 Diabetes and diabetic kidney disease: a comparatively developed evidence cluster

### 3.1 Why diabetes/DKD is a broad current reference module

Among the four modules reviewed, diabetes and DKD form a comparatively developed translational cluster. This does not mean that diabetes AI is broadly deployment-ready. It means that this module contains a wider range of clinically relevant signals than the other disease modules: evidence synthesis, non-invasive triage, externally validated kidney and cardiovascular risk prediction, calibration and decision-curve reporting, prospective or near-workflow decision-support studies, and explicit cautionary examples where transportability or calibration remains problematic (10, 20, 21).

The module is also clinically coherent. Diabetes care already depends on repeated measurement, risk stratification, treatment adjustment, screening, referral, and complication surveillance. DKD and diabetic retinopathy, in particular, create decision points where earlier identification or more precise triage could plausibly affect follow-up intensity, referral timing, or preventive management. This makes diabetes/DKD a useful test case for the review’s central claim: actionability depends on connecting a prediction to a decision, not merely improving an algorithmic metric.

### 3.2 DKD risk prediction and non-invasive triage

The DKD literature includes both broad model-development evidence and more clinically instructive examples. Review-level evidence on machine-learning risk prediction for DKD and diabetic nephropathy helps establish that this is a crowded and active field, but it also reinforces the need for careful validation and bias appraisal before models are treated as clinically reliable (10, 22).

More clinically instructive examples move beyond a single development dataset. Retinal-image deep learning and retinal vascular parameters combined with clinical variables illustrate how accessible ocular data can be connected to clinically meaningful DKD triage questions, although these approaches still require careful attention to external validation, workflow placement, and downstream consequences (20, 23). Clinlabomics-based occult DKD screening and advanced-DKD prediction studies add examples in which SHAP, calibration, DCA, and web-based or clinician-facing outputs begin to link model estimates with screening or risk-stratification decisions (24, 25).

One deployment-oriented DKD model is especially important for this module because it combines several actionability elements in one study. The PSMMC NephraRisk model was developed in a large T2D registry, reported discrimination and calibration, used decision-curve analysis to estimate threshold-based net benefit, included subgroup fairness assessment, and implemented a clinician-facing web application (19). These features make it a useful DKD anchor among the reviewed papers, but they do not prove cross-system transportability or patient-outcome benefit without broader external and prospective evaluation.

Other DKD papers sharpen the same point from different angles. A Korean multi-hospital EHR study is valuable because it shows that simpler models can remain competitive under external validation, reminding readers that algorithmic complexity is not itself an actionability criterion (26). Metabolite-based DN prediction with SHAP adds a biomarker and explanation angle, but its single-center nature means it should be framed as mechanistically suggestive and technically informative rather than deployment-ready (27).

### 3.3 Diabetes decision support and workflow evidence

The diabetes module also contains the clearest evidence that AI can enter clinical decision workflows. A real-time AI-assisted insulin titration system tested in a randomized clinical-trial setting is one of the clearest examples that the evidence base is moving beyond retrospective prediction, while multicenter hypoglycemia-risk prediction illustrates a related inpatient safety task that still needs prospective workflow testing (28, 29). A reviewer-suggested gestational-diabetes model broadens this cluster to pregnancy-specific endocrine prediction: it reported 96.4% accuracy, an ROC-AUC of 0.982, and a 28% false-positive-rate reduction using multi-cohort data, but this should be interpreted as algorithmic prediction evidence rather than proof of prospective workflow benefit (30). The importance of this contrast is not that all endocrine AI should be judged against the same intervention, but that treatment-facing models require evaluation at the point where a recommendation may change care.

An integrated primary-care diabetes model adds a different kind of actionability evidence by combining image-based deep learning and language-model components. Its value lies in workflow-level evaluation: the model is evaluated as part of a care pathway involving primary-care detection, referral, and patient-management signals, rather than as a classifier alone (31). A diabetic-retinopathy implementation framework reinforces the same point from another diabetes complication: validation, prospective comparison, head-to-head testing, and cost-effectiveness matter before screening AI is treated as health-system ready (32). Together, these examples contrast with studies that report a web interface or explanation layer without testing how clinicians or patients actually use the output.

Older decision-support concepts remain useful, but they should be used sparingly. A three-stage intelligent-support framework and earlier diabetes decision-support reviews support the idea that trust, validity, explainability, and user-facing workflow have long been recognized as implementation problems (14, 33, 34). Current diabetes/DKD claims should still rely primarily on recent, task-specific, and full-text-verified evidence rather than historical background reviews.

### 3.4 Calibration, transportability, and cautionary evidence

The diabetes/DKD module is useful partly because it contains its own warnings. External validation does not guarantee transportability if calibration degrades or local recalibration is needed. A DKD mortality model developed from the MIMIC database and a Chinese cohort supports the caution that externally evaluated risk models may retain discrimination while requiring careful calibration assessment before local action (35). This is the kind of evidence that prevents the review from becoming a simple catalogue of high-performing models.

Low-cost or routine-data models also need careful framing. Routine blood and biochemical DKD screening models may be attractive because their predictors are accessible, but modest performance or limited validation can constrain clinical actionability (36). Vision-threatening diabetic retinopathy, pediatric T1D complication-risk, and interpretable diabetes-classification studies add useful examples of XAI and clinician-facing risk classification, yet they remain limited if they do not demonstrate robust prospective workflow effects, fairness, or monitoring (37–39).

### 3.5 What the diabetes/DKD module teaches

The diabetes/DKD evidence suggests three lessons for endocrine AI as a whole. First, actionability is strengthened when model outputs are connected to a clinical task such as screening, triage, referral, or treatment adjustment. Second, calibration, decision-curve analysis, and external validation often matter more for decision support than small differences in algorithm class. Third, even comparatively mature modules still have major gaps in cross-system prospective validation, subgroup fairness, post-deployment monitoring, and proof of patient benefit. Diabetes/DKD therefore serves as both the clearest evidence cluster and the clearest reminder that XAI is only one component of clinical actionability.

## 4 Thyroid disease: utility metrics are improving, but implementation evidence remains incomplete

### 4.1 Thyroid nodule and cancer-risk prediction

Thyroid disease provides a useful contrast to diabetes/DKD because many studies are closer to diagnostic triage than to longitudinal disease management. The most actionable thyroid papers are not simply those with the highest AUC, but those that connect malignancy, metastasis, biopsy, or surgical-risk prediction to calibration, threshold-based utility, interpretable predictors, and clinically recognizable outputs. External validation of a thyroid cancer risk model with decision-curve analysis is an important example because it makes calibration limits visible while still estimating net benefit across plausible decision thresholds (40).

For thyroid nodules, more informative studies begin to link imaging or clinical prediction to biopsy or diagnostic triage. A multicenter CT-based explainable thyroid-nodule model reported high internal and external discrimination, calibration, DCA, a nomogram, and SHAP interpretation, making it a lead example of thyroid AI moving beyond pure classification (41). Thy-Wise is also action-linked because it compared an ultrasound-only model against American College of Radiology Thyroid Imaging Reporting and Data System (ACR TI-RADS) and reported improved specificity with fewer unnecessary fine-needle aspiration biopsy (FNAB) recommendations while preserving sensitivity (42). These examples are clinically more informative than another isolated high-AUC classifier because they address the decision that thyroid clinicians actually face: whether a nodule should undergo further investigation.

The thyroid module also shows why actionability should not be equated with technical sophistication. Multimodal, cytological, and radiomics models can add useful information, but their clinical value depends on validation design and decision linkage. A low-resource multimodal thyroid-nodule model using CLIP-derived ultrasound features illustrates accessible AI development, but its modest discrimination and lack of external validation make it support evidence rather than a maturity anchor (43). A multimodal cytology deep-learning model points to another clinically relevant workflow, but it should still be framed as diagnostic-support evidence rather than proof of deployed decision support (44). Similarly, intratumoral and peritumoral ultrasound radiomics with SHAP supports the promise of interpretable thyroid imaging, but test performance and single-center design require cautious wording (45).

### 4.2 Surgical risk, metastasis, and recurrence tools

Papillary thyroid cancer studies extend thyroid AI from nodule classification into surgical and advanced-risk decisions. Models for central lymph node metastasis and distant metastasis are clinically relevant because their outputs could influence preoperative counseling, surgical planning, or surveillance intensity. A CLNM model with temporal external validation, SHAP, calibration, and DCA supports this action-linked framing, although the retrospective design and small external cohort prevent deployment-ready claims (46).

A multicenter distant-metastasis model is a stronger thyroid cancer example because it combines external testing, SHAP interpretation, calibration/DCA, and a web calculator for distant metastasis risk (47). A paratracheal lymph-node metastasis model adds external testing, SHAP, and web-calculator support, while also showing transportability limits when external performance drops (48). Together, these studies show that thyroid AI can support surgical-risk stratification only when the output is tied to a recognizable action and when external validation is reported transparently.

Recurrence prediction further illustrates the difference between a tool prototype and clinical actionability. The TC check web application reports very high discrimination, SHAP explanations, and a clinician-facing interface, which makes it useful as a model-to-tool translation example (49). However, without broader external validation, prospective use, or evidence that the tool changes surveillance decisions safely, it should be described as a promising prototype rather than a validated recurrence-management system.

### 4.3 Treatment, appropriateness, and clinician-facing explanation

Thyroid AI is not limited to imaging. Graves’ hyperthyroidism provides a treatment-decision example in which an interpretable model estimated remission after initial radioactive iodine therapy and used SHAP, Brier score, and DCA to connect prediction with treatment planning (50). The study is valuable because the predicted outcome is directly action-linked, but its retrospective single-center design means that it should not be generalized to routine treatment selection without external and prospective validation.

Other thyroid papers point toward workflow actionability rather than individual diagnosis. NLP-based identification of thyroid ultrasound appropriateness supports population-level evaluation of overuse and documentation patterns, which is a different but clinically meaningful actionability pathway (51). A dual-center thyroid-nodule screening model based on accessible demographic and biochemical indicators also suggests a low-cost triage direction, although the outcome is nodule presence rather than malignancy or patient benefit (52). A reader-assistance XAI framework adds a different angle by comparing structured feature explainability with Grad-CAM and reporting improved physician performance, but it remains a reader-support study rather than a deployment trial (53).

### 4.4 What the thyroid module teaches

The thyroid evidence supports a middle position in the actionability ladder. Compared with many early endocrine AI studies, thyroid papers more often report external validation, calibration, DCA, SHAP, nomograms, web calculators, biopsy-triage logic, or workflow appropriateness tools. Compared with selected diabetes/DKD examples, however, the module still lacks prospective implementation studies, post-deployment monitoring, and clear evidence that AI-supported decisions improve patient outcomes. Thyroid AI is therefore best framed as an emerging clinical-utility module: more developed than a technical imaging catalogue, but not yet a fully implemented decision-support field.

## 5 PCOS and the PMOS terminology transition: from interpretable diagnosis to endocrine-metabolic workflow support

### 5.1 Terminology and clinical framing

The condition historically and still most commonly called polycystic ovary syndrome (PCOS) has recently been proposed to be renamed polyendocrine metabolic ovarian syndrome (PMOS), reflecting its endocrine, metabolic, reproductive, psychological, and dermatological dimensions rather than a narrow focus on ovarian cysts (9). This terminology matters for an AI review because model labels can shape what data are collected, what outcomes are predicted, and whether algorithms prioritize ovarian morphology alone or broader endocrine-metabolic risk. Because most AI papers, datasets, and source titles still use PCOS, this review uses PCOS as the default term and refers to PMOS when discussing the proposed terminology transition or the broader multisystem framing.

The PCOS/PMOS module should be written as an actionability problem, not as a catalogue of impressive diagnostic scores. Review evidence on PCOS diagnostic AI and detection techniques shows frequent reports of high performance alongside persistent limits from small samples, class imbalance, heterogeneous datasets, low uptake of XAI, and insufficient external validation (11, 54). A broader ovarian-AI review similarly warns that high apparent performance can coexist with heterogeneity, retrospective bias, and implementation or governance gaps (55). These review-level cautions are essential because several individual studies report very high or even perfect performance metrics that would be risky to present without validation skepticism.

### 5.2 Diagnostic prediction and endocrine-metabolic risk stratification

A multicenter explainable machine-learning and nomogram study is the most informative current PCOS/PMOS actionability source identified in this review. It developed a model for early detection and risk stratification, reported internal and external AUCs, used calibration and clinical impact/decision-curve analysis, and applied SHAP to identify influential endocrine-metabolic predictors such as DHEAS, AMH, triglycerides, and age (56). These features make it a better-supported actionability example than a performance-only classifier, but the retrospective design and geographically bounded validation still require caution.

Other diagnostic studies illustrate both promise and risk. A multi-stack XAI framework and a clinical-ultrasound model demonstrate how SHAP, LIME, feature importance, and other interpretability tools can make PCOS/PMOS prediction more inspectable (57). At the same time, extremely high or perfect external metrics in some diagnostic models should be treated as a reason for deeper validation scrutiny rather than as evidence of immediate clinical readiness (58). In this module, unusually high performance is not a shortcut to actionability; it is a reason to examine leakage risk, dataset representativeness, calibration, and external reproducibility.

Ultrasound AI provides a clinically intuitive bridge between ovarian morphology and workflow support. A YOLOv11-based ultrasound model reported high internal and external validation performance for automatic recognition and follicle-counting support, suggesting a route toward reducing operator burden in ovarian ultrasound assessment (59). Yet its actionability remains limited unless future studies show how the output affects diagnostic pathways, clinician time, patient communication, or decisions about endocrine-metabolic evaluation beyond morphology.

### 5.3 EHR prediction, reproductive outcomes, and clinical translation

EHR-based PCOS/PMOS prediction is especially relevant to actionability because diagnostic delay is a real clinical problem. A machine-learning study using electronic health records predicted PCOS-related outcomes before clinical diagnosis, with gradient boosting performing well across several model formulations (60). Its value is not that it establishes a deployable screening rule, but that it points to a plausible workflow: earlier recognition of patients who may need endocrine-reproductive evaluation. The current limitation is that this evidence comes from a single health system and lacks external hospital validation, prospective CDS testing, and fairness or subgroup calibration. Additional EHR and disparity evidence makes the equity issue concrete: missed PCOS diagnosis was associated with race/ethnicity, insurance, and social vulnerability patterns, while review-level evidence shows racial and ethnic variation in phenotype and metabolic burden that current datasets often underrepresent (61, 62).

PCOS/PMOS also intersects with reproductive decision support. An XGBoost model for live birth after fresh embryo transfer in IVF/ICSI patients with PCOS supports the idea that AI may eventually assist counseling or treatment planning beyond diagnosis (63). However, this evidence should be framed as reproductive-prognosis support rather than a validated clinical decision rule, because the study lacks external IVF-center validation and does not show that AI-supported counseling improves outcomes.

The key interpretive discipline in this module is to avoid converting prediction into clinical translation by tone alone. PCOS/PMOS AI currently contains promising diagnostic, ultrasound, EHR, reproductive-prognosis, and diagnostic-equity examples, but the evidence is still sparse for prospective workflow testing, subgroup calibration, multi-health-system fairness evaluation, and post-deployment monitoring. Review-level cautions and the best-supported current examples should therefore carry the argument, rather than historical background alone.

### 5.4 What the PCOS/PMOS module teaches

The PCOS/PMOS module teaches that terminology, phenotype breadth, and data design are part of actionability. If AI systems are trained only to reproduce historical PCOS labels or ovarian-morphology patterns, they may miss the multisystem endocrine-metabolic framing now emphasized by PMOS terminology. The more clinically useful direction is not merely earlier classification, but earlier recognition linked to endocrine-metabolic risk assessment, reproductive counseling, workflow triage, and equitable follow-up. At present, the evidence supports cautious optimism, not deployment confidence.

## 6 Obesity and GLP-1 response heterogeneity: personalization is promising but still early

### 6.1 GLP-1 response heterogeneity as the clinical problem

The obesity/GLP-1 module differs from the other disease sections because the clinical action is often treatment personalization over time rather than one-time diagnosis or triage. Real-world registry evidence shows substantial heterogeneity after GLP-1 receptor agonist initiation: only a minority achieved both HbA1c and weight targets, while many achieved only one target or neither (64). This finding is not AI evidence, but it establishes the clinical need for predictive tools that distinguish glycemic from weight response and, in future work, incorporate adherence, tolerability, and long-term maintenance. Real-world semaglutide/tirzepatide data further show why this task cannot be reduced to response prediction alone: persistence and dose-titration adherence were only moderate, and stronger weight-loss estimates came from patients who remained persistent (65).

For clarity, this section distinguishes direct AI evidence for incretin-response or treatment-benefit prediction from contextual or adjacent evidence on response heterogeneity, persistence, tolerability, digital support programs, remote-care workflows, and broader obesity-risk modeling.

The current AI evidence for GLP-1 response is more concrete than a general personalization claim, but still early. A real-world data study of GLP-1 pathway drug responsiveness found that logistic regression outperformed tested machine-learning models for HbA1c response prediction, with baseline HbA1c emerging as a dominant predictor (66). This is useful because it cautions against assuming that complex algorithms automatically improve actionability. More recent examples strengthen the response-prediction layer: an AI-driven analysis combining cardiovascular-outcome trial data with real-world data identified externally validated subgroups with different glucagon-like peptide-1 receptor agonist (GLP-1RA) cardiovascular-benefit profiles (67), while a digitally collected semaglutide support-program dataset showed that a trial-derived exposure-response model could predict 26- and 52-week categorical weight-loss response with low bias (68). An explainable ML study of 69,194 GLP-1RA initiators also identified phenotypes associated with early treatment intensification, but intensification is a proxy outcome that can reflect therapeutic inertia and the study did not test prospective CDS (69). A systematic review and integration framework for AI-enabled semaglutide personalization and a T2D drug-response AI review further map potential inputs and treatment-response tasks while emphasizing longer follow-up, explainability, regulatory validation, and clinical evaluation before routine implementation (5, 70).

These sources justify a cautious claim: GLP-1 personalization is an important research frontier, and direct response or benefit prediction is beginning to appear, but it is not yet a mature endocrine AI module. The field still needs prospectively tested treatment-selection pathways for modern incretin and dual-agonist cohorts, clear decision thresholds, calibration, net-benefit evaluation, safety and tolerability prediction, adherence monitoring, fairness analysis, and evidence that AI-supported selection improves outcomes. Remote semaglutide/tirzepatide program evidence adds useful workflow and tolerability context, but it remains observational program evidence rather than AI-guided drug-selection proof (71).

### 6.2 Personalized weight management beyond drug response

Broader obesity AI provides a richer, but less drug-specific, evidence base. A review of AI across the obesity continuum supports the idea that obesity management can integrate mechanistic insights, prevention, treatment-response prediction, digital therapeutics, and population-health tools, while emphasizing explainability, fairness, privacy, data quality, and monitoring (4). This review-level source helps frame the module, but it should not be used as proof that any particular obesity AI system improves clinical outcomes.

Original obesity and metabolic-risk studies show several adjacent actionability pathways. IoT-based personalized childhood weight-management AI uses lifestyle data and SHAP explanations to generate individualized weight-management guidance and reports an external-validation performance drop that is still clinically informative (72). A sarcopenic-obesity model based on readily available clinical features provides an example of accessible XAI risk stratification with cross-setting validation and visible transportability decline (73). Multiomics and XAI for pediatric insulin-resistance prediction adds a longitudinal prevention angle, but its small sample size and lack of independent external validation require conservative wording (74).

These papers support personalized obesity management in a broader sense, but they should not be merged with GLP-1 treatment-response evidence. A useful separation is clinical heterogeneity in GLP-1 response, early AI/real-world data (RWD) prediction for drug response, and adjacent obesity/metabolic XAI tools that may support lifestyle, prevention, or risk-stratification decisions.

### 6.3 Public-health screening, lifestyle explanation, and transferability

Several obesity-related studies are useful mainly because they reveal the gap between high internal metrics and real-world actionability. A LightGBM model for adolescent metabolic syndrome reported high internal performance and an online deployment, but internal validation with class-imbalance handling is not enough to establish external clinical utility (75). A Bi-LSTM/LIME obesity-level classifier illustrates individualized lifestyle and anthropometric explanations, yet remains limited by internal evaluation and dataset-specific generalizability (76).

An explainable clinical decision-support model for large-for-gestational-age births among women with overweight or obesity is particularly helpful as a cautionary actionability example because external validation revealed weaker generalization and population-sensitive performance (77). This aligns with the review’s broader framework: obesity and metabolic-risk models often operate across heterogeneous populations, so subgroup calibration, fairness, and monitoring are not optional extras. They are central to deciding whether a model can safely guide action.

### 6.4 What the obesity/GLP-1 module teaches

The obesity/GLP-1 module requires particularly careful tone. The rationale for personalization is strong, and early AI/RWD evidence now includes response prediction, benefit stratification, treatment-intensification phenotyping, and digital feedback-loop examples. However, GLP-1 treatment selection remains less developed than diabetes/DKD and selected thyroid examples because prospective workflow testing, monitoring, and outcome evidence are still limited. The defensible conclusion is conditional: obesity AI can support risk stratification, lifestyle personalization, and emerging response or benefit prediction, but GLP-1 treatment selection should not yet be framed as established clinical decision support.

## 7 Qualitative evidence map and practical appraisal questions

**Table 2**, **Figure 2**, and **Supplementary Table 1** provide complementary levels of synthesis. **Table 2** presents one concise actionability anchor and the main unresolved gap for each disease module. **Figure 2** presents a qualitative maturity matrix across six actionability domains. **Supplementary Table 1** adds a study-level comparative layer, showing how representative studies differ in reported performance metrics, validation design, calibration, DCA, XAI, workflow evidence, fairness or subgroup assessment, monitoring, and key limitations. Their purpose is comparative appraisal rather than formal scoring: they indicate where the reviewed evidence is comparatively broader, emerging, or limited across the actionability domains defined in Section 2. In this synthesis, limited means sparse, indirect, or not yet tested evidence rather than evidence absence.

**Table 2:** Concise cross-module evidence map for endocrine AI actionability.

Module anchor	Key gap
Diabetes/DKD. Prospective or near-workflow decision support, multicenter risk prediction, and integrated primary-care models provide the broadest cross-module actionability anchors (28, 29, 31)	Multisite prospective outcome evaluation, subgroup calibration, evidence of patient benefit, and post-deployment monitoring.
Thyroid disease. Externally validated nodule and biopsy-triage models increasingly report calibration or DCA and clinician-facing tools (41, 42)	Prospective workflow and patient-outcome testing, fairness assessment, and post-deployment monitoring.
PCOS/PMOS. Multicenter explainable risk stratification and emerging ultrasound or EHR workflow support provide early actionability anchors (56, 59, 60)	Phenotype alignment during the PMOS transition, multi-health-system validation, subgroup fairness, and prospective workflow testing.
Obesity/GLP-1. Real-world response prediction and treatment-intensification phenotyping provide early treatment-oriented anchors (66, 69)	Comparative treatment-effect evidence, calibrated benefit-harm thresholds, adherence and tolerability modeling, prospective decision-policy evaluation, and monitoring.

This table condenses the comparison into one actionability anchor and one principal gap for each disease module. It is intended as a synthesis table rather than as a complete study inventory.

The synthesis should therefore be read as a qualitative comparison of actionability domains, not as a numerical ranking. Diabetes/DKD has comparatively broader evidence across several checks; thyroid disease is more developed for validation and utility than for workflow implementation; PCOS/PMOS and obesity/GLP-1 are clinically important but earlier for fairness, monitoring, and prospective decision-support testing. A recurring pattern across the source studies is that high discrimination or accuracy can coexist with limited transportability, missing action thresholds, sparse subgroup analysis, or absent post-deployment plans.

This domain-by-domain comparison is the appropriate form of rigor for a narrative, framework-oriented Review. It does not test new ablations or generate new statistical significance results; instead, it isolates which actionability requirements are met, partially met, or not reported across representative studies and clinical contexts. The result is a deeper cross-module synthesis than a disease-by-disease inventory and a clearer explanation of why technically strong models may still remain clinically preliminary.

## 8 Discussion: actionability gates, not headline discrimination metrics

### 8.1 Clinical task linkage is the first dividing line

Across modules, the first useful comparison is not whether a model reports a high AUC, but whether the predicted output is tied to a clinical task that could plausibly change care. Diabetes/DKD has the clearest task linkage because its models address screening, triage, complication surveillance, insulin titration, inpatient safety, and workflow support. The randomized insulin-titration study and the primary-care diabetes workflow model show that some diabetes AI is already being evaluated in or near care pathways rather than only in retrospective datasets (28, 31). Hypoglycemia prediction and diabetic-retinopathy implementation frameworks broaden this task linkage, but they also show why prospective workflow and health-system evaluation remain necessary (29, 32).

Thyroid studies also have relatively clear action targets, especially biopsy reduction, surgical-risk stratification, and treatment-response prediction. Thy-Wise links prediction to FNAB triage, while distant-metastasis and thyroid-nodule models use external validation, DCA, and clinician-facing tools to connect risk estimates to recognizable endocrine decisions (42, 47). In PCOS/PMOS, the task link is emerging through early detection, ultrasound workflow support, and reproductive-prognosis prediction, but these links remain less often tested in prospective clinical workflows (56, 60). In obesity/GLP-1, the clinical need for personalization is strong and direct AI evidence now includes response, benefit, and treatment-intensification prediction examples (66–68). A newer GLP-1RA intensification study and broader T2D drug-response AI synthesis sharpen the treatment-action problem, but prospective drug-response decision support remains early (69, 70).

### 8.2 Reliability checks separate better-supported examples from performance-only models

The second comparison is reliability. Transparent reporting and bias/applicability appraisal define whether a study can be judged, while calibration and DCA test whether model output is useful for threshold-based decisions (12, 13). Diabetes/DKD and thyroid contain the most concrete examples: a DKD model reports calibration, DCA, fairness analysis, and a web application, while selected thyroid studies report external validation, calibration/DCA, SHAP, and clinician-facing outputs (19, 41, 47). These examples are better supported than studies that present discrimination and feature importance alone.

PCOS/PMOS has at least one clinically informative diagnostic-risk example. The multicenter explainable nomogram study combines external validation, calibration, clinical impact or decision-curve analysis, SHAP, and a nomogram, making it a useful PCOS/PMOS anchor for this review (56). However, many PCOS papers still require caution because high apparent performance can be affected by dataset size, class imbalance, leakage, phenotype heterogeneity, and limited external validation (11, 55). Obesity/GLP-1 is improving on reliability checks for response or benefit prediction: one GLP-1RA study adds external validation for treatment-benefit stratification, and one semaglutide study adds digitally updated weight-response prediction. However, neither establishes prospective AI-guided treatment selection, and another real-world response-prediction study cautions that simpler clinical models may outperform more complex machine-learning approaches in some settings (66–68).

### 8.3 XAI is common, but workflow proof is rare

XAI methods appear across all modules, but they do not carry the same actionability weight in each disease area. In thyroid ultrasound, feature explainability and reader-assistance studies can make image-based models more clinically inspectable (53). In personalized weight management, SHAP can connect lifestyle or IoT features to individualized guidance (72). In PCOS/PMOS, DKD, and broader diabetes prediction, SHAP or other feature-attribution methods can identify endocrine-metabolic, imaging, or biomarker predictors that appear clinically plausible (27, 39, 56).

The cross-module lesson is that explainability is an inspection layer, not a clinical endpoint. An explanation may increase transparency, reveal implausible predictors, or help a clinician understand a risk score, but it does not prove improved decisions, better outcomes, or safer implementation. Early clinical evaluation and workflow-fit questions therefore need their own evidence: Who receives the output, when, in what format, against what comparator, and with what safeguards against overreliance or drift (6, 14).

### 8.4 Fairness and monitoring remain underdeveloped shared checks

Fairness and post-deployment monitoring remain the least developed translation checks across the disease modules. Methodologically, these checks are not optional because endocrine AI may behave differently by sex, age, ethnicity, BMI, disease subtype, laboratory platform, imaging device, medication access, socioeconomic context, or care setting (17, 18). Empirically, however, the extracted endocrine papers more often report discrimination, validation, calibration, DCA, or XAI than explicit subgroup calibration, fairness, drift monitoring, or post-deployment surveillance.

A DKD deployment-oriented study is a useful exception because it reports subgroup fairness analysis alongside calibration, DCA, and a web application (19). The obesity-related large-for-gestational-age CDSS is valuable for a different reason: it shows how performance can be population-sensitive, making transportability and equity concerns visible (77). PCOS/PMOS raises additional fairness questions because the proposed PMOS framing emphasizes multisystem endocrine-metabolic features, while many historical datasets still reflect PCOS-era labels, referral patterns, and phenotype definitions (9, 11). Recent PCOS disparity evidence gives this concern a concrete clinical analogue: diagnostic recognition and phenotype description vary by race/ethnicity, insurance, social vulnerability, and representation patterns, so fairness should be assessed before or during implementation rather than postponed until after deployment (61, 62). These gaps should shape the review’s recommendations: model development is only the first part of translation; governance and monitoring determine whether action remains safe over time.

Privacy-preserving and federated approaches offer one route to the multi-institutional collaboration that external validation and post-deployment monitoring require. For example, a federated, homomorphically encrypted diabetic-retinopathy model has been proposed to enable cross-site training without sharing raw patient data (78). Such designs are promising for governance and scalability, but the reported performance remains largely internal, reinforcing that strong in-sample accuracy does not by itself establish external transportability or clinical actionability.

### 8.5 Qualitative maturity synthesis

**Figure 2** summarizes this comparison as a qualitative maturity matrix rather than a formal score. Diabetes/DKD is comparatively broader across clinical task linkage, validation, calibration or decision-curve reporting, and workflow evidence, while fairness is still emerging and monitoring remains limited. Thyroid disease is comparatively broader for task linkage, validation, and clinical-utility reporting, but workflow, fairness, and monitoring evidence remain less developed. PCOS/PMOS and obesity/GLP-1 are not absent of evidence; they are earlier in different ways, and domains marked limited indicate sparse, indirect, or not yet tested evidence. The imbalance in section depth therefore reflects the uneven maturity of the underlying evidence rather than a claim that some endocrine domains are intrinsically more important. PCOS/PMOS needs broader phenotype alignment, equity-aware validation, and workflow testing, whereas obesity/GLP-1 needs prospective treatment-selection evaluation, adherence and tolerability modeling, fairness analysis, and monitoring before routine clinical decision support can be claimed.

## 9 Practical recommendations and future directions

The following recommendations are practical appraisal suggestions for authors, reviewers, editors, clinicians, and health systems. They are not formal clinical guidelines or disease-specific practice recommendations.

### 9.1 Recommendations for authors

Authors developing endocrine AI models should begin by naming the clinical decision, not the algorithm. A manuscript should state whether the model is intended for screening, diagnosis, triage, treatment selection, dose adjustment, surveillance, or monitoring; who will use it; what action may change; and what harm could occur if the output is wrong. This task-first framing is essential because a model for thyroid biopsy triage, DKD screening, PCOS/PMOS early recognition, and GLP-1 response prediction faces different thresholds, data constraints, and safety risks.

Reporting and bias appraisal should be treated as entry gates. TRIPOD+AI can structure reporting of prediction models, while PROBAST+AI can guide risk-of-bias and applicability appraisal for regression or AI-based prediction models (12, 13). Authors should describe patient selection, predictor availability, outcome definitions, missing-data handling, model-development steps, validation design, and intended-use setting clearly enough for readers to judge whether the model could be transported to their own practice.

Performance reporting should move beyond discrimination. Calibration should be reported whenever predictions may guide absolute-risk or threshold-based decisions, and DCA should be used when plausible threshold probabilities can be defined (7, 8). Disease examples show why this matters: DKD and thyroid studies that report calibration, DCA, and external validation provide more actionable evidence than performance-only models (19, 41). Authors should also report whether a model remains useful after local recalibration, across external sites, or in clinically meaningful subgroups.

XAI should be linked to a purpose. SHAP, LIME, Grad-CAM, feature explainability, nomograms, or web calculators should clarify a decision, expose model behavior, support error analysis, or improve communication. They should not be presented as stand-alone evidence of safety or benefit. If an explanation is intended to support clinician decisions, authors should test whether users understand it, whether it changes decisions appropriately, and whether it creates automation bias (6, 53).

Finally, authors should describe implementation assumptions. A deployable endocrine AI paper should specify the data pipeline, timing of output, responsible user, alert format, fail-safe plan, monitoring metric, retraining or recalibration trigger, and fairness analysis. This is especially important for PCOS/PMOS and obesity/GLP-1, where phenotype heterogeneity, treatment access, lifestyle context, and longitudinal response patterns can amplify bias or drift (17, 18).

### 9.2 Recommendations for reviewers and editors

Reviewers should ask whether the strength of the claim matches the strength of the evidence. A retrospective internally validated model may support technical feasibility, but it should not be described as clinically actionable without external validation, calibration, threshold-based utility, and workflow evidence. A model with XAI can be more inspectable than a black box, but explanation does not replace early clinical evaluation or implementation testing (6, 14).

Reviewers should also separate three levels of evidence. The first is model-development evidence: predictors, discrimination, internal validation, and explanation. The second is reliability evidence: external or temporal validation, calibration, DCA, subgroup performance, and applicability appraisal. The third is implementation evidence: workflow testing, clinician interaction, patient-management consequences, monitoring, fairness, and safety. Diabetes/DKD currently has examples across more of these levels, including fairness-aware DKD prediction and insulin titration (19, 28), as well as primary-care workflow and retinopathy implementation work (31, 32). PCOS/PMOS and obesity/GLP-1 more often remain between the first and second levels, including PCOS diagnostic-risk modeling (56) and GLP-1 response or benefit prediction (66–68).

For very high or perfect performance values, reviewers should be more cautious, not less. They should examine sample size, class balance, leakage, preprocessing, feature availability, duplicate patients, external validation, and whether the test set resembles the intended-use population. This is especially relevant in PCOS/PMOS diagnostic models and obesity or metabolic-risk classifiers, where dataset specificity can make performance look more stable than it will be in practice (58, 77).

Editors and reviewers could strengthen the field by asking endocrine AI submissions to include an actionability summary table. Such a table could report intended clinical action, data source, validation type, calibration, DCA or threshold analysis, explanation method, workflow setting, fairness/subgroup analysis, monitoring plan, and current evidence level. This would make it harder for manuscripts to imply clinical readiness from AUC and XAI alone, while still allowing early-stage models to be published with transparent evidence boundaries.

### 9.3 Recommendations for clinicians and health systems

Clinicians should interpret endocrine AI tools as decision-support instruments, not autonomous clinical rules. Before trusting a model, the practical questions are straightforward: Was it validated in a population like mine? Are the predictors available at the moment of decision? Is the risk estimate calibrated locally? Is there a clear threshold for action? Does using the tool improve or safely change the workflow? Are subgroup performance and monitoring available?

For diabetes/DKD, these questions can identify the difference between promising risk prediction and genuinely useful screening, triage, or treatment-support systems. For thyroid disease, they help distinguish biopsy or surgical-risk tools with external validation and DCA from high-AUC imaging models without workflow testing. For PCOS/PMOS, they keep diagnostic-delay and ultrasound support tools connected to broader endocrine-metabolic assessment rather than narrow historical labels. For obesity/GLP-1, they prevent early response-prediction evidence from being overused as treatment-selection proof.

Health systems considering endocrine AI should plan monitoring before deployment. Monitoring should include performance, calibration, alert burden, missingness, subgroup behavior, clinician override, patient consequences, and drift after changes in assays, imaging devices, EHR templates, medication patterns, or referral pathways (18). Fairness should be evaluated before and after deployment because endocrine disease expression, diagnostic access, treatment access, and follow-up intensity vary across populations (17). Without this infrastructure, even a well-explained model can become unsafe or inequitable as the care environment changes.

For real-time implementation, endocrine AI models must define when the prediction is generated, what predictors are available at that exact moment, who receives the output, what threshold triggers action, what comparator workflow is being improved, how clinician override is handled, and how calibration drift and subgroup performance will be monitored over time. Adjacent work outside endocrinology illustrates both the appeal and the limits of real-time deployment: an AI-based critical-care framework pairs a random-forest classifier with ICD-10-linked treatment suggestions and a continuously refreshing remote dashboard (79). Yet such systems are often trained and evaluated on synthetic or retrospective data, so a deployable-looking real-time interface still does not demonstrate prospective workflow safety or benefit. Endocrine AI faces the same implementation gap.

### 9.4 Future directions

The next phase of endocrine AI research should prioritize prospective, task-specific evaluation over adding more retrospective high-AUC models. Diabetes/DKD should move from well-studied prediction and early CDS evidence toward multicenter prospective outcome studies, subgroup calibration, and post-deployment monitoring. Thyroid AI should test whether biopsy, surgical-risk, recurrence, and treatment-response tools actually change clinician decisions and patient pathways safely. PCOS/PMOS research should adapt to the PMOS terminology transition by modeling multisystem endocrine-metabolic phenotypes, diagnostic delay, reproductive outcomes, and psychological dimensions (9, 11). Fairness across diverse populations should be evaluated explicitly rather than assumed from aggregate performance (61, 62).

Recent obesity/GLP-1 examples define a cautious research agenda: AI-driven subgrouping can identify GLP-1RA benefit profiles, digitally collected data can support semaglutide weight-response prediction over follow-up, and explainable ML can identify phenotypes associated with early GLP-1RA treatment intensification (67–69). Real-world semaglutide/tirzepatide persistence and remote-program data add the equally important care-process layer, but they are observational and should not be treated as AI-guided treatment-selection evidence (65, 71). Current evidence mainly characterizes heterogeneity or predicts response under observed therapy rather than estimating comparative benefit and harm across realistic treatment or management options (64, 66, 70).

Before AI-guided GLP-1 treatment selection is trialled, the minimum pretrial evidence package should therefore include external and temporal validation, calibrated estimates for weight, glycemic, tolerability, and persistence outcomes, prespecified action thresholds, net-benefit comparison with simple clinical rules, and subgroup safety assessment. A locked decision policy could then be evaluated prospectively for its effects on treatment choice and patient-centered outcomes.

Across all modules, the most important future direction is not more elaborate explanation alone. The field needs evaluation designs that connect explanation to decisions, decisions to workflow, workflow to outcomes, and outcomes to monitoring. A clinically actionable endocrine AI model is therefore not the model with the largest AUC or the most colorful explanation plot. It is the model whose output can be interpreted, trusted, thresholded, acted on, monitored, and revised within the realities of endocrine care.

### 9.5 Review limitations

This review has limitations. It is narrative and framework-oriented rather than systematic, so study identification and weighting were purposive and oriented toward clinical actionability rather than exhaustive coverage. It did not perform formal meta-analysis or quantitative scoring. The cross-module maturity labels are qualitative and based on the reviewed evidence; they should not be interpreted as formal scores or as rankings of all endocrine AI domains. Direct comparisons are also constrained by heterogeneity in outcomes, datasets, and implementation contexts, particularly in PCOS/PMOS and obesity/GLP-1 studies. Finally, fairness, subgroup calibration, prospective workflow evidence, and post-deployment monitoring remain sparsely reported across the literature, which limits how confidently translational maturity can be inferred.

## 10 Conclusion

Endocrine AI will become clinically useful only when prediction and explanation are connected to decisions that can be evaluated, acted on, monitored, and revised. Across the four modules reviewed here, the main differences lie in how far evidence travels from prediction to workflow, fairness, and monitoring, not in algorithms or AUC values alone. Diabetes/DKD currently has a comparatively broad evidence base, thyroid disease shows increasingly detailed validation and clinical-utility reporting, PCOS/PMOS has high clinical need but remains constrained by phenotype, fairness, and workflow gaps during the PMOS transition, and obesity/GLP-1 management is beginning to gain direct response, benefit, and treatment-intensification evidence while still needing prospective treatment-selection and monitoring studies.

The actionability framework helps prevent two common overclaims: treating explainability as proof of clinical utility, and treating high retrospective performance as evidence of patient benefit. Numerical model results are useful only when interpreted beside translational evidence. **Supplementary Table 1** supports this interpretation by presenting performance metrics alongside translational checks, rather than as a stand-alone ranking. The Review has limitations: it is narrative and framework-oriented rather than systematic, the maturity labels are qualitative appraisal aids rather than formal scores, and direct cross-module comparison is constrained by heterogeneity in outcomes, datasets, and implementation contexts.

The field should move from retrospective explanation toward prospective, task-specific, and workflow-facing evaluation. Models should specify the clinical action, report and appraise data and modeling transparently, validate and calibrate across settings, evaluate threshold-based utility, compare against simpler clinical rules when appropriate, test workflow effects, assess fairness, and plan monitoring before deployment. The practical goal is AI support that can inform endocrine decisions without obscuring uncertainty, widening inequities, or drifting silently after implementation.
