# Supplementary material for: From explainability to clinical actionability: translating artificial intelligence models into decision support for endocrine disease management
Source: Front Endocrinol (Lausanne). 2026 Aug 21;17:1887643. doi: 10.3389/fendo.2026.1887643 (PMC13542687; doi:10.3389/fendo.2026.1887643)
Supplement: Supplementary file 1 [file Table1.docx]

Supplementary Table S1. Study-level comparative matrix for endocrine AI actionability

This matrix is a structured narrative-synthesis aid. NR means not reported or not clearly reported in the source study as reviewed for this manuscript. It is not a formal risk-of-bias tool, meta-analysis, or validated scoring system.

| Study / ref. | Module | Clinical task | Reported performance | Design | Validation | Calibration | DCA / net benefit | XAI / interpretability | Workflow / usability | Fairness / subgroup | Monitoring | Key limitation |
| --- | --- | --- | --- | --- | --- | --- | --- | --- | --- | --- | --- | --- |
| Tourkmani et al. (19) | Diabetes/DKD | 3-year DKD risk prediction | Discrimination plus calibration/DCA reported | Development/validation/deployment-oriented clinical model | Internal plus deployment-oriented evaluation | Reported | Reported | Ensemble/model interpretation; web application | Web application / implementation signal | Subgroup fairness reported | Limited post-deployment outcome monitoring | Strong anchor but still requires multicenter prospective outcome evidence |
| Meng et al. (20) | Diabetes/DKD | Non-invasive DKD biopsy/triage using retinal images | Population-based model performance reported | Population-based imaging study | Validation reported | NR | NR | Deep learning imaging output | Triage relevance, not routine workflow trial | NR | NR | Patient-benefit evidence remains indirect |
| Ying et al. (28) | Diabetes care | Real-time AI-assisted insulin titration | Clinical trial outcomes reported | Randomized clinical trial | Prospective clinical evaluation | NR | NR | Decision-support protocol rather than XAI emphasis | Evaluated in care pathway | Limited/NR | NR | Generalizability and monitoring require further work |
| Li et al. (31) | Diabetes care | Primary-care diabetes detection/referral support | Clinical workflow performance reported | Integrated image/language-model workflow study | Validation reported | Limited/NR | NR | Image/language model outputs | Primary-care workflow signal | Limited/NR | NR | Needs broader prospective health-system evaluation |
| Muthuselvi et al. (30) | Gestational diabetes / diabetes care | Early-stage GDM prediction | Accuracy 96.4%, ROC-AUC 0.982, and 28% false-positive-rate reduction reported | Random-forest predictive model using multi-cohort data | Cross-cohort and pooled validation reported | Limited/NR | NR | Feature selection and random-forest feature importance | No prospective workflow test | Cross-population generalizability addressed; subgroup fairness NR | NR | Pregnancy-specific endocrine prediction example; high reported performance does not establish workflow benefit or patient outcomes |
| Jayalakshmi and Tamilvizhi (78) | Diabetic retinopathy / privacy-preserving diabetes AI | Federated DR detection | Accuracy 98.6%, precision 98.8%, recall 98.3%, F-score 98.6%, specificity 98.1% reported | Federated learning with homomorphic encryption and client selection | Primarily internal/experimental evaluation | NR | NR | Deep/federated architecture, not clinician-facing XAI | No prospective workflow test | Privacy-preserving design; subgroup fairness NR | NR | Promising governance architecture but high internal performance does not establish clinical actionability |
| Fernandez Alba et al. (40) | Thyroid | Thyroid cancer risk prediction / biopsy triage | External validation and DCA reported | External validation study | External validation | Reported/assessed | Reported | Clinical risk framing | Biopsy-triage relevance | NR | NR | Good utility example but not a workflow-outcome trial |
| He et al. (41) | Thyroid | Benign vs malignant thyroid nodule prediction | Multicenter performance reported | Multicenter CT-based explainable ML | Multicenter validation | Reported/partial | Reported/partial | SHAP / explainable ML | Clinician-facing interpretability signal | NR | NR | Implementation and outcome effects remain untested |
| Jin et al. (42) | Thyroid | Thyroid nodule evaluation and FNAB support | Model performance and triage implications reported | Interpretable ML model | Validation reported | Reported/partial | Clinical triage/utility addressed | Interpretable model / tool | FNAB triage relevance | NR | NR | Needs broader prospective workflow evidence |
| Hou et al. (47) | Thyroid | Distant metastasis prediction in papillary thyroid cancer | Validation, calibration/DCA, and calculator reported | Multicenter prediction study | External/multicenter validation | Reported | Reported | SHAP / calculator | Web calculator prototype | NR | NR | Web tool is an implementation signal, not proof of patient benefit |
| Lu et al. (50) | Thyroid | RAI therapy optimization in Graves hyperthyroidism | Performance, Brier score, and DCA reported | Interpretable ML treatment-planning study | Validation reported | Brier/calibration-related evidence reported | Reported | SHAP | Treatment-planning relevance | NR | NR | Prospective treatment-decision evaluation is still needed |
| Yao et al. (56) | PCOS/PMOS | Early detection and risk stratification of PCOS | Discrimination, calibration, DCA/CIC, SHAP, and nomogram reported | Multicenter explainable model and nomogram | External/multicenter validation | Reported | Reported | SHAP / nomogram | Diagnostic risk stratification signal | Limited/NR | NR | Phenotype and health-system transportability remain concerns |
| Zad et al. (60) | PCOS/PMOS | EHR-based PCOS prediction | Gradient-boosting performance reported across formulations | EHR prediction study | Internal/retrospective validation | Limited/NR | NR | Model-based feature signals | Potential early-detection workflow | Representation concerns remain | NR | Needs prospective diagnostic-delay and workflow evaluation |
| Morieri et al. (67) | Obesity/GLP-1 / T2D | GLP-1RA cardiovascular-benefit stratification | AI-driven subgroup/benefit stratification reported | Multisource CVOT and real-world data analysis | External/multisource evidence | Limited/NR | Benefit stratification rather than standard DCA | AI-driven subgrouping | Treatment-benefit framing | Subgrouping central to study | NR | Does not establish routine AI-guided treatment selection |
| Faerch et al. (68) | Obesity/GLP-1 | Semaglutide weight-response prediction over follow-up | 26- and 52-week categorical response prediction reported with low bias | Digitally collected real-world data | Validation reported | Bias/calibration-related evidence reported | NR | Prediction model | Digital follow-up context | Limited/NR | NR | Observational program data; adherence and missingness can shape estimates |
| Falcetta et al. (69) | Obesity/GLP-1 / T2D | Early GLP-1RA treatment intensification | Explainable ML predictors reported in 69,194 initiators | Real-world ML study | Validation reported | Limited/NR | NR | Explainable ML / SHAP | Treatment-intensification phenotype signal | Limited/NR | NR | Outcome reflects care-process decisions, not direct response |
| Richards et al. (71) | Obesity/GLP-1 | Remote semaglutide/tirzepatide care process | Persistence and weight-loss outcomes reported | Retrospective observational remote program | Not an AI model validation study | NR | NR | NR | Remote-care workflow context | NR | NR | Implementation context but not AI-guided selection evidence |
| Tamilvizhi et al. (79) | Adjacent CDS example; critical care, not endocrine evidence | Real-time monitoring and treatment recommendation prototype | 94.2% accuracy on 10,000 simulated ICU samples reported | Random-forest prototype with ICD-10 and Streamlit dashboard | Synthetic/retrospective-style evaluation | NR | NR | Random forest; interface prototype | Real-time dashboard / remote access prototype | NR | NR | Adjacent example showing that a real-time interface does not prove prospective workflow safety or benefit |
